# Supplementary figures and images for: Immune response against Chlamydia trachomatis via toll-like receptors is negatively regulated by SIGIRR
Source: PLoS One. 2020 Mar 25;15(3):e0230718. doi: 10.1371/journal.pone.0230718 (PMC7094862; doi:10.1371/journal.pone.0230718)

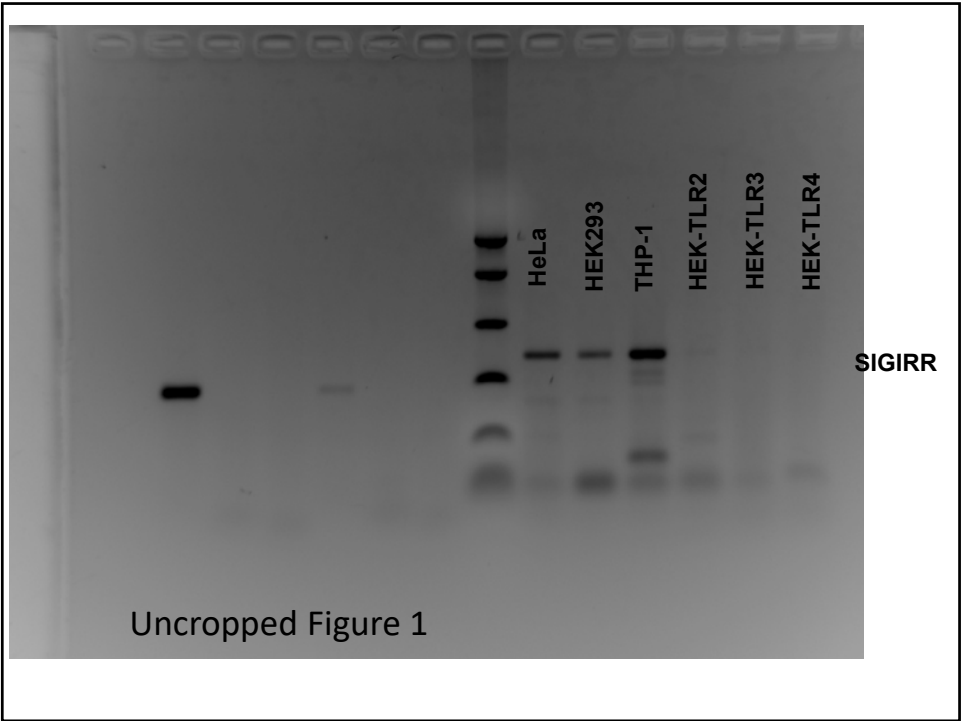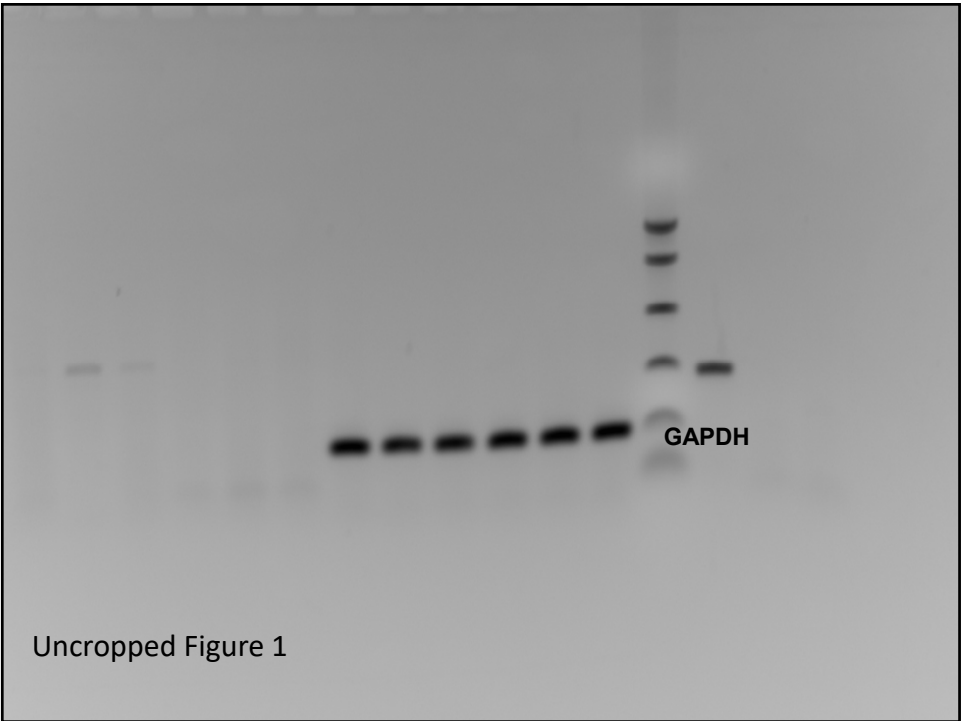

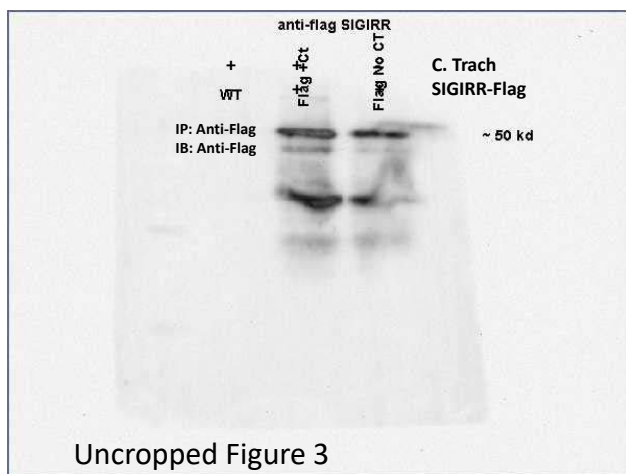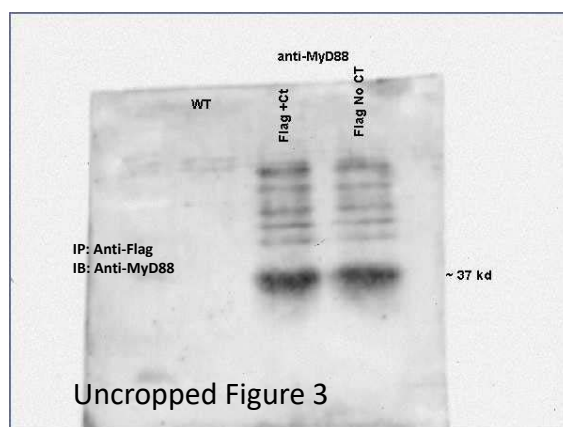

Supplement: S1 Raw images — (PDF) [file pone.0230718.s001.pdf]
